# Establishing Composition of Solid Solution Based on Single Crystal and Powder X-ray Measurement: The Case of Halogenated Bismuth(III) Complexes with Acetophenone-4-methyl-3-thiosemicarbazone

**DOI:** 10.3390/ijms251910814

**Published:** 2024-10-08

**Authors:** Anita M. Grześkiewicz, Grzegorz Dutkiewicz, Ibrahim I. Ozturk, Maciej Kubicki

**Affiliations:** 1 Faculty of Chemistry, Adam Mickiewicz University, 61-614 Poznań, Poland; 2 Section of Inorganic Chemistry, Department of Chemistry, Tekirdag Namık Kemal University, Tekirdag 59030, Türkiye

**Keywords:** solid solutions, X-ray diffraction (XRD), Bi complexes, Vegard’s rule

## Abstract

New bismuth (III) complexes with acetophenone-4-methyl-3-thiosemicarbazone (L) and halogens (Cl and Br) in both bridging and terminal positions have been synthesized and structurally characterized using single-crystal X-ray diffraction. The pure complexes (Cl or Br) were found to be highly isostructural, which motivated our attempts to create solid solutions of these complexes. A series of such compounds was prepared using various procedures and stoichiometries. A method for determining the mutual concentrations of different halogens, based on the positions of selected peaks in powder diffraction patterns, was tested and compared with other methods.

## 1. Introduction

Substitutional solid solutions, which may be roughly defined as two-component crystals in which one of the components (minor, solute) occupies the sites in a stochastic manner, defined by the crystal lattice of the other (major, solvent). However, there is also another kind of solid solution, interstitial solution, in which the solute occupies the positions between the lattice sites (for instance, steel may be regarded as such a solution of carbon in iron). Historically, solid solutions—mainly in the form of metal alloys, e.g., bronze—have played a very important role in the development of human civilization, and for a long time, metallurgy was a main field of research on such substances. In the first quarter of the twentieth century, some empirical rules (Hume-Rothery’s rules [1,2,3]) were formulated, describing the optimal conditions for creating substitutional solid solutions (similar atomic radii, differing by no more than 15%, similar electronegativity, crystal structures, etc.) as well as interstitial solutions (e.g., solute atoms should have radii smaller than 59% of those of solvent atoms). Not so long after, an essential title describing solid solutions of organic compounds written by Kitaigorodsky was published [4]. Since then, solid solutions have also become an active field of research [5,6,7,8], and the reasons for this are of dual origins; the first is technological development, resulting in much easier and more precise experimental determination of the crystal structure (more potent radiation sources, better detectors, more powerful computers, etc.), and the second reason is the importance of such research for practical applications, for instance, in the pharmacological industry or technology. This research is related to the search for new polymorphic forms and more effective crystallization techniques (e.g., [9,10,11,12,13,14]). Recently, the number of papers focused on solid solutions of organic compounds has increased quite quickly. This research was systematized and summarized in the review by M. Lusi [15]. One of the important issues in such research is a determination of the concentration of the two components of the solution, which is especially interesting and important in the organic solid solution where the solubility can be infinite, i.e., one can meet all concentrations, from 0 to 100%. If single crystals of appropriate quality are available, the solution is relatively simple, as the X-ray structure determination allows for refining the occupancy factors of both components. However, such a situation is rather unusual, and some other methods may be used. One of the easiest and most available methods is powder X-ray diffraction (PXRD). Metallurgy, for over 100 years, has utilized the so-called Vegard’s law, or rather Vegard’s rule (as it is rarely closely obeyed), an empirical finding stating that the lattice parameters of the solid solution of two constituents are approximately a weighted mean of the lattice parameters of the constituents, with the weights reflecting relative concentrations of both components [16,17]. We decided to compare this rule with single-crystal occupancy results and others based on monitoring certain peaks in the powder diffractogram for a quite complex system: a pair of the two-centred Bi complexes with Br and Cl substituents in both bridging and terminal positions. It might be noted that such solid solutions (Br–Cl pair) are relatively popular (e.g., [18,19]). This strategy allows for a number of possible combinations of the solutes; in principle, we are dealing with many-component crystals in this case. Thus, the aim of this study was to prepare series of new, solid solutions of bismuth(III) complexes with thiosemicarbazones, which differ in Cl/Br stoichiometry and establish their composition using different crystallographic approaches.

## 2. Results and Discussion

**Scheme 1:**
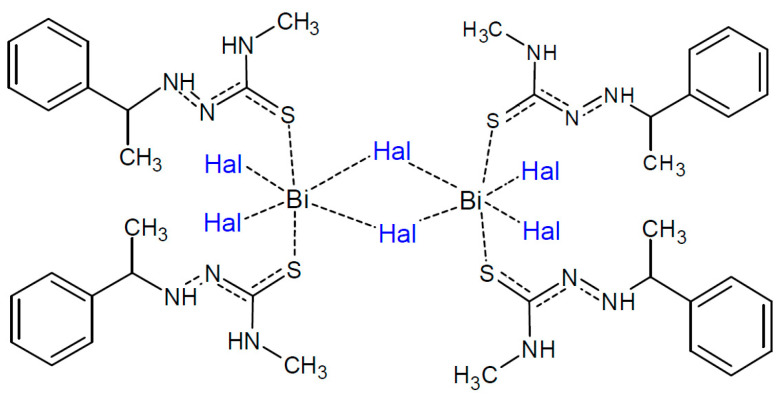
Structural formula of complexes **1** and **2**, where Hal corresponds to given halogen (Cl or Br).

**Figure 1:**
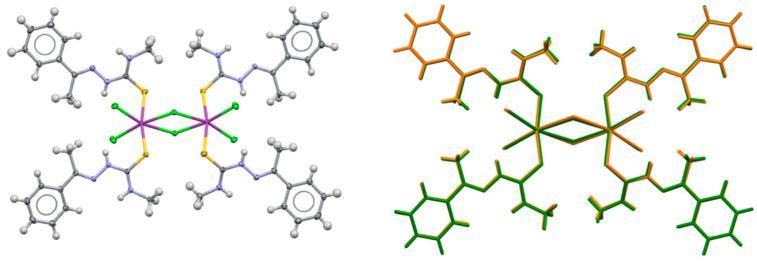
(**left**) Perspective view of the complex **1**. The ellipsoids are drawn at the 50% probability level, hydrogen atoms are shown as spheres of arbitrary radii, (**right**) Superposition of complexes **1** (green) and **2** (orange) with bismuth–halogen part fitted. The geometrical parameters have been included in SM (Table S1).

In the course of our studies on bismuth complexes, we obtained two new complexes of bismuth (III) with acetophenone-4-methyl-3-thiosemicarbazone (L) and halogens (Cl (**1**) and Br (**2**), cf. Scheme 1, Figure 1). As these complexes turned out to be highly isostructural, we attempted to obtain solid solutions of them. Six structures of such systems were determined by single-crystal X-ray diffraction (**1**a, **1**b, **1**c, **2**a, **2**b, **2**c).

### 2.1. Structures of the Complexes

Both complexes are two-centered, with the general formula bis(μ_2_-X)Bi_2_X_4_L_4_ (X = Cl in **1**, Br in **2**) and trans-disposition of ligand molecules (Figure 1). The components are C_i_ symmetrical, with the center of inversion at the midpoint of the Bi···Bi distance. The coordination of Bi(III) centers can be described as distorted octahedral centers (cf. Table S1—Supplementary Materials (SM) for relevant geometrical parameters).

The complexes described here are highly isostructural; the degree of this can be described, for instance, by parameters such as the unit cell similarity index and isostructurality index [20]. The values of these parameters are 0.010 and 99.25%, respectively, very close to ideal values of 0 and 100%, which proves the very high similarity of the crystal structures (cf. Figure 1, right).

### 2.2. Solid Solutions

Due to the chemical and structural similarities between molecules **1** and **2**, it is highly likely that solid solutions can form between these complexes. Specifically, there are 64 possible combinations of Cl and Br atoms across the six available positions, though molecular symmetry reduces this number. There are three primary centers of halogen exchange: two terminal and one bridging (Figure S1, SM). Single-crystal X-ray diffraction (SCXRD) provides occupancies of Cl/Br atoms at each position, but these reflect only average values and do not reveal the exact ratios of molecules within the crystal. However, SCXRD does allow for an estimation of the Cl/Br proportion at each halogen site in the unit cell, offering far more information than methods like differential scanning calorimetry (DSC) or Vegard’s law.

To obtain solid solutions, we prepared mixtures with molar ratios of 1:2 (a), 1:1 (b), and 2:1 (c) by adding an exchanging halogen anion to the dissolved complex (full procedure described in Section 3). Using both complexes **1** and **2** as starting materials, we generated a total of six solid solutions (**1**a, **1**b, **1**c, **2**a, **2**b, **2**c). The number preceding the letter refers to the starting material (1 for complex **1** and 2 for complex **2**), while the letter indicates the molar ratio of the solution from which the crystal was obtained (a = 1:2, b = 1:1, c = 2:1). For example, **1**a corresponds to complex **1** with a 1:2 ratio of Br-, while **2**c represents complex **2** with a 2:1 ratio of Cl^−^. This dual approach aimed to determine whether structural differences would arise depending on the starting material used for crystallization.

For each crystallization trial, single-crystal X-ray diffraction (SCXRD) was performed on at least two crystals to assess potential differences in composition within the same batch. The stoichiometric ratio of components was determined based on occupancy factors (OF) for alternative atoms, and sample homogeneity was further verified by powder X-ray diffraction (PXRD). The results were consistent, with no differences greater than one percent in the average composition for most trials. Considering that most crystals exhibited twinning, this level of consistency is quite remarkable. The only exceptions were **2**a and **2**b, where two distinct crystal forms—plates and needles—were identified, which will be discussed in more detail later.

**Figure 2:**
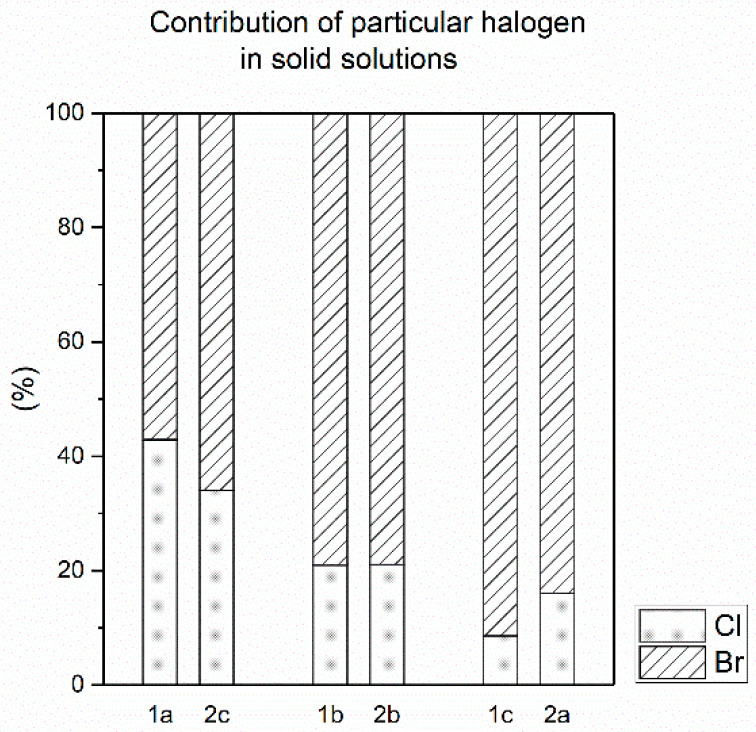
Contribution of particular halogen atoms in solid solution estimated based on SCXRD.

The occupancies were established independently for each of three available halogen positions (the values for each are listed in Table S2, SM). However, when comparing the results with those of other methods, the average content of each halogen in the solid solution is more informative, so the mean values of the OF values determined for three halogen atoms have been calculated and used (Table 1). The results in Table 1 and Figure 2 and Figure S2 (SM) show that there is a strong preference to form solid solutions with the predominance of bromine, even in the case of a significant 2:1 prevalence of chlorine in the preparative solution. Next, the comparison of appropriate values in Table S2 shows that the terminal atoms undergo substitution more easily than the bridging atoms. Comparing different ways of theoretically obtaining the same Br:Cl ratio, it is evident that the most similar values of the substitution have been obtained in a 1:1 molar ratio of Cl/Br (Br/Cl). The average percentage Cl:Br ratio is 21:79 (≈1:4) (Figure 2), and very similar results are obtained regardless of which complex was used as the starting one. Such a comparison is not so simple in other cases of 1:2 and 2:1 Cl/Br proportions in the starting solutions. When the starting solution is that of complex **1** and Br^−^ is added in a molar ratio of 2:1, the proportion Cl:Br in the final solid solution is 43:57, but when one starts from complex 2 and Cl^−^ is added in a molar ratio of 1:2, this proportion is 34:66. This may indicate the relative stability of the complexes in solution.

**Figure 3:**
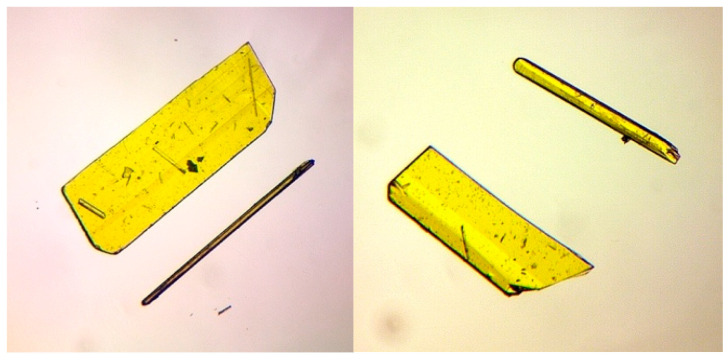
The photography of different crystal types in **2**a (**left**) and **2**b (**right**) solid solutions.

Quite unexpectedly, two distinct crystal morphologies were observed in the cases of **2**a and **2**b. Both plates and needles appeared within the same crystallization batch (Figure 3), with the differences being more pronounced in **2**a. SCXRD analysis was performed separately for each form, with at least two crystals of each type examined. In both cases, the space groups, molecular arrangements, and complex structures were identical, suggesting that the observed differences occur at a level other than the crystal structure itself.

It was determined that the needle forms are non-merohedral twins, while the plates are clearly single crystals, with over 98% of reflections indexed to a single-domain unit cell. A second, more subtle difference was found in the occupancy factors of **2**a. In the needle form (**2**a_n), the Cl:Br ratio was 18(1):82(1), while in the plate form (**2**a_p), it was 15(1):85(1). Although these differences are close to three e.s.d., they were consistently observed across different samples.

For **2**b, the comparison was inconclusive, possibly due to the poor quality of the **2**b_n crystals. In this case, the largest difference in calculated occupancies between crystals from the same batch was 1.54%, and varying refinement strategies could alter the calculated occupancies by up to one percent. As a result, only for **2**a will we distinguish between the two crystal forms throughout the remainder of the article. Nevertheless, the average occupancy in **2**b was the same as in **2**b_p, so for comparison purposes, we will rely on the more precise data obtained from **2**b_p.

### 2.3. Vegard’s Law

According to Vegard’s law, at constant temperature, there is a linear relationship between the lattice parameters of an alloy (or other solid solution) and the concentrations of its constituent elements [16,17]. Therefore, if the parameters for the pure forms of an alloy are known, it should be possible to determine the composition of any solid solution based solely on the unit cell parameters.

**Figure 4:**
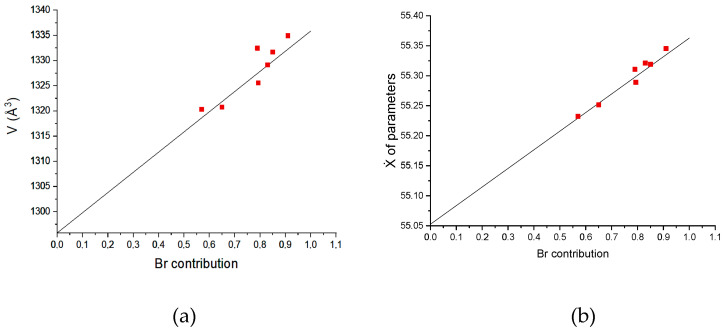
Graphical representation of the deviation from Vegard’s law calculated for (**a**) unit cell volumes and (**b**) the average of occupancies calculated for each unit cell parameter (a, b, c, α, β, γ), separately.

We applied Vegard’s law to estimate the Cl/Br ratio in the solid solutions using unit cell parameters obtained from SCXRD. The selected comparison of unit cell parameters from the SCXRD analysis is presented in Table 1, while all individual data are available in Table S3 (SM), and graphical representations are shown in Figure 4 and Figure S2 (SM). However, depending on the parameter used, discrepancies in the results were observed, sometimes as high as 28%. The most questionable results came from the *c*_0_ parameter of the unit cell, where the discrepancy between Cl/Br molar ratios determined from SCXRD occupancies and those predicted by Vegard’s law reached an average of 13%. This may be attributed to the minimal difference in the *c*_0_ parameter between pure complexes **1** and **2** (only 0.5%).

It is generally accepted that Vegard’s law is more reliably applied using parameters that account for all unit cell dimensions. Therefore, in Table 1, we included:

the volume of the unit cell (with an average discrepancy of 8%),
occupancy calculated from the average value of all unit cell parameters (with a discrepancy of 6% on average),
and the average of occupancies calculated for each unit cell parameter separately ($\bar{X}$), where the most coherent results were observed in comparison to SCXRD occupancy factors (with an average difference of 3%).

**Figure 5:**
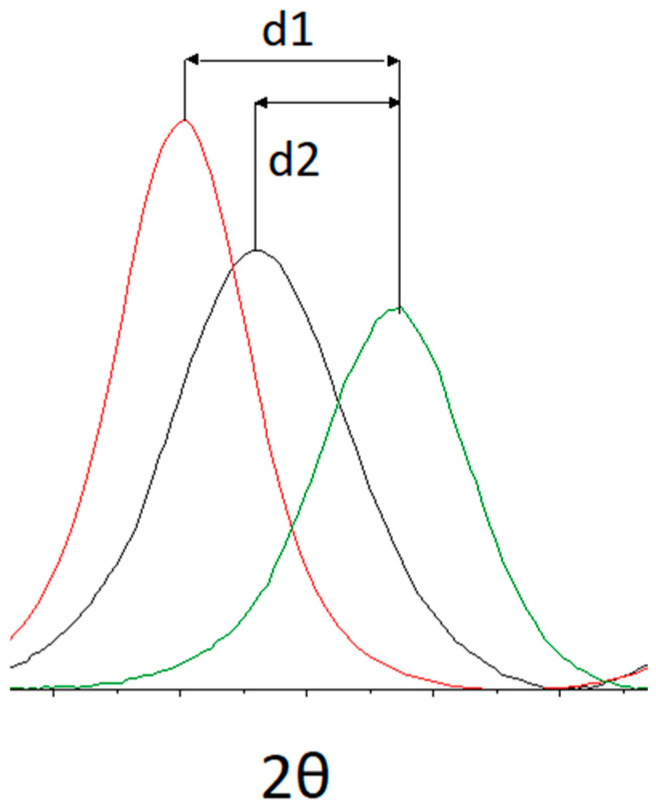
Descriptors of the solid solution establishment (red profile is from complex **2**, green from complex **1**, and the black one from the solid solution).

These observations are clearly visible in the graph in Figure 5, where the black line represents the bromine concentration in the solid solution calculated using Vegard’s law (based on the parameters from complexes **1** and **2**). The red line corresponds to the results obtained from single-crystal studies of solid solutions (**1**a–c, **2**a–c, Br_contribution ≈ occupancy factors). The experimentally determined concentrations are closest to Vegard’s law prediction when using $\bar{X}$.

### 2.4. Powder Data

The conclusions of our analysis so far can be summarized as follows: if the unit cell parameters are accurately determined, it is possible to obtain a reasonably good approximation of the molar ratio in a solid solution, even in a complex system with three independent halogen exchange centers. This can be performed by averaging occupancies or using the unit cell volume, as demonstrated by Adams et al. [18]. However, an important question arises: how do we proceed when obtaining precise unit cell dimensions is difficult or nearly impossible?

A related parameter is the position of peaks in the powder diffraction pattern, typically expressed as the 2θ angle. Isostructural complexes generally exhibit similar, though not identical, diffractograms, with peak shifts occurring between those of the pure forms (Figure 5). This well-known phenomenon can be used to estimate the concentration of one component. Hill et al. [7], for example, employed a single peak to confirm concentrations. However, for a system as complex as the one we are describing, selecting a single representative signal is almost impossible.

A more rational approach is to select multiple reflections and calculate an average value based on peak shifts. While this sounds straightforward, it presents challenges due to limitations we must impose to obtain reliable data. In principle, identifying a few well-defined, equivalent reflections that meet specific criteria should allow us to estimate the molar ratio of the solid solution based on the 2θ angle positions.

Implemented restraints:

The net intensity of the reflection should be significant.

The distance between neighboring reflections should be more than 0.1° (i.e., reflections must be well separated).

The difference between the signal positions of the pure complexes **1** and **2** should exceed 0.15° (this value can be lowered if the data resolution is very high).

These three criteria are essential for obtaining reliable data. If the intensity is too low, accurately determining the reflection position becomes difficult. On the other hand, even with strong reflections, if they are too close to each other, the ability to determine the exact shift decreases dramatically. Similarly, small shifts between derivatives complicate precise measurement.

During our study, four reflections (0.1-1; 100; 10-1; 10-2) that met these criteria were selected. The differences between the signals from the solid solutions and the pure forms were compared. Figure 5 defines the parameters used to estimate the molar ratio of the solid solutions. The contribution of the Br atom in the solid solution is calculated as (d2/d1) × 100%.

Initially, theoretical powder diffractograms were generated to assess how accurately the molar ratios could be determined using a limited number of selected reflections and to compare these results with experimental data. The theoretical and experimental PXRD data are presented in Table S4 and Figure S3 (SM). In all cases, the peaks from the solid solutions are shifted closer to the signal from complex **2** (Br) than to complex **1**, which—at least qualitatively—agrees with the SCXRD results.

**Figure 6:**
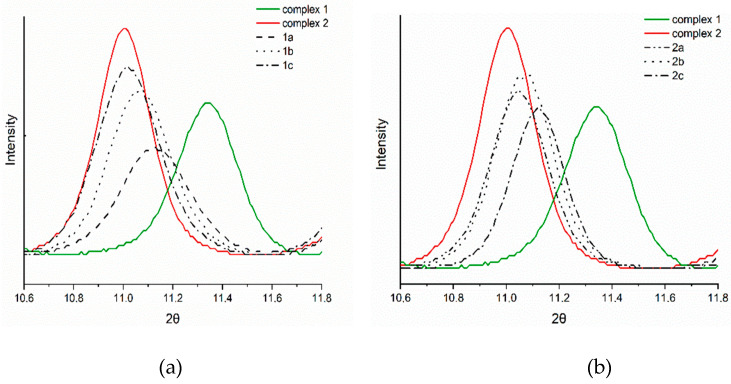
The position of the 10-1 reflex in complexes **1** and **2** and solid solutions (**a**) **1**a–c, and (**b**) **2**a–c (experimental data). The 2θ angle is given in °.

Moreover, the magnitude of the shifts correlates well with the bromine content in the crystals. The most leftward shift is observed for **1**c and **2**a, followed by **1**b, **2**b, and finally **1**a and **2**c (Figure 6, Figure S3 SM). Additionally, for **1**b and **2**b, the calculated occupancies based on SCXRD are identical, and the selected reflections in the powder diffractograms of these samples appear at the same 2θ values. The 2θ angle is measured in degrees (°).

The results of the bromine and chlorine contributions in the solid solutions, based on differences in peak positions, are summarized in Table 1. Surprisingly, these values are more accurate than expected, considering that only a small portion of the data (4 out of more than 20 reflections) was used for the calculations. Even more compelling is that these values are closer to the SCXRD-based data than those obtained using Vegard’s law, whether calculated based on unit cell volume or the average occupancies from all unit cell parameters.

The highest discrepancy in the experimental data is observed for compound 1a, but even then, the error remains below 10%, while the average difference is less than 5%, which is quite satisfactory compared to other approaches (as summarized in Table 1). Interestingly, and perhaps predictably, the theoretical and experimental powder diffraction patterns are similar, with the largest difference being 5% for 2b. We also analyzed the bromine concentration based on each individual reflection (Table S5, SM), but the results were less consistent than those calculated from the average.

## 3. Materials and Methods

### 3.1. Synthesis

The acetophenone 4-methyl-3-thiosemicarbazide, bismuth (III) chloride, bismuth (III) bromide, and all solvents were purchased commercially and used as received.

Acetophenone-4-methyl-3-thiosemicarbazone was synthesized according to the literature procedure by the reflux method [21,22].

Complex **1**: 0.5 mmol of bismuth (III) chloride (158 mg) was dissolved in 10 mL of ethanol and then a few drops of conc. HCl were added. Then, 1.0 mmol 1-phenylethyl-N-methylthiosemicarbazide (207.3 mg) was dissolved in 10 mL of ethanol and added slowly to the above solution. The yellow solution that formed was refluxed for 3 h and cooled to room temperature. The clear solution was filtered and allowed to evaporate slowly at room temperature until product was formed. After a few days crystals suitable for X-ray diffraction analysis were obtained.

Complex **2**: 0.25 mmol of bismuth (III) bromide (112 mg) was dissolved in 10 mL of methanol. Then, 0.5 mmol acetophenone-4-methyl-3-thiosemicarbazone (104 mg) was dissolved in 10 mL of methanol and added slowly to the above solution. The yellow solution that formed was refluxed for 3 h and cooled to room temperature. The clear solution was filtered and kept in the refrigerator overnight at 8^°^ to obtain crystals suitable for X-ray diffraction analysis.

**1**a–c: 0.3 mmol of {[BiCl_2_(μ_2_-Cl)(η^1^-S-Hacpmtsc)_2_]_2_} (43.8 mg) was weighed into each of three round bottom flasks and stirred in 15 mL of methanol until completely dissolved. To the solutions, 0.9 mmol (~10.40 μL), 1.8 mmol (~20.80 μL), and 3.6 mmol (~41.59 μL) of hydrobromic acid (47%) were added, respectively. The clear solutions were filtered and kept in the refrigerator for several days and then the products were collected.

**2**a–c: 0.3 mmol of {[BiBr_2_(μ_2_-Br)(η^1^-S-Hacpmtsc)_2_]_2_} (51.8 mg) was weighed into each of three round bottom flasks and stirred in 15 mL of methanol until completely dissolved. To the solutions, 0.9 mmol (~7.45 μL), 1.8 mmol (~14.90 μL), and 3.6 mmol (~29.80 μL) of hydrochloric acid (37%) were added, respectively. The clear solutions were filtered and kept in the refrigerator for several days and then the products were collected.

### 3.2. Single-Crystal X-ray Measurement

X-ray diffraction data were collected by the ω-scan technique on a four-circle Rigaku Xcalibur diffractometer equipped with an Eos detector equipped with a graphite-monochromized MoKα radiation source (λ = 0.71073 Å) [23] in 100 K. The data were corrected for Lorentz polarization effects as well as for absorption [24]. Accurate unit cell parameters were determined by a least-squares fit of the reflections of the highest intensity, which were chosen from the whole experiment. The calculations were mainly performed within the OLEX2 [25]. The structures were solved with ShelxT [26] and refined with the full-matrix least-squares procedure on F^2^ by SHELXL-2015/2017 [27]. Scattering factors incorporated in SHELXL were used. All non-hydrogen atoms were refined anisotropically, and hydrogen atoms were located at the calculated positions and refined as a “riding model” with isotropic thermal parameters fixed at 1.2 times the U_eq_ of the appropriate carrier atom for compounds. The structure **2**a_n has been refined as a twin with the hklf5 file. The structure representation has been prepared with Mercury software ver. 2022.3.8 [28]. Crystallographic data and refinement details are listed in Table 2.

### 3.3. Powder X-ray Measurement

Powder X-ray studies were performed with an Agilent Technologies SuperNova diffractometer with monochromated CuKα radiation (λ = 1.54178 Å) at room temperature. The 2θ range for the measurement was 0°–50° with 30 s exposure time. Diffractograms were analyzed with the KDif v.3.01b program from the Kalvados package [29].

## 4. Conclusions

The comparison of various methods for determining the Cl/Br molar ratio in a series of halogenated Bi (III) complexes with acetophenone-4-methyl-3-thiosemicarbazone demonstrates that all approaches can yield reasonable results when appropriate limitations are applied. For Vegard’s law, the best match with SCXRD data was obtained by averaging the occupancies calculated for each unit cell parameter (a, b, c, α, β, γ) separately. Surprisingly, comparable results were also achieved using PXRD diffractograms, despite relying on only four carefully selected reflections. This is promising, as PXRD is far less time-consuming and demanding than methods like Vegard’s law.

PXRD offers several advantages, including not requiring unit cell parameters and being largely non-destructive, unlike thermal methods. Moreover, PXRD can help confirm or exclude polymorphism and provide immediate information about sample homogeneity. However, it has limitations, such as the need for crystallinity and the fact that it only provides averaged results for complex systems. Nonetheless, in this study—where three halogen exchange centers are possible—it produced highly accurate results, making it a valuable method for qualitative studies of solid solutions.

## Figures and Tables

**Table 1:** Bromine concentration in solid solutions calculated based on different approaches. ^a^ The average of occupancies calculated for each parameter separately (a, b, c, α, β, γ); ^b^ Unit cell volume in [Å^3^]; ^c^ The occupancy calculated from average value of all unit cell parameters (a, b, c, α, β, γ).

		Bromine Concentration in Solid Solutions Calculated Based on:					
No	Cl/Br	SCXRD	Vegard’s Law			PXRD	
			Av ^a^	V ^b^	Oc ^c^	Theory	Experimental
**1** **a**	2:1	57.3(6)	57.79(2)	65.2(1)	62.93(2)	65.47(1)	66.05(2)
**1** **b**	1:1	79(2)	78.17(2)	78.2(3)	82.01(2)	77.26(1)	81.02(2)
**1** **c**	1:2	92.1(9)	97.15(2)	101.78(8)	99.59(2)	95.04(1)	97.13(2)
	Br/Cl						
**2** **a**	2:1	84(2)	85.46(2)	90.5(2)	89.10(2)	88.20(1)	89.55(2)
**2** **b**	1:1	79(1)	84.45(2)	95.6(1)	88.15(2)	85.86(1)	81.02(2)
**2** **c**	1:2	65.5(6)	62.01(2)	66.33(8)	69.18(2)	66.37(1)	69.14(2)

**Table 2:** Crystal data, data collection, and structure refinement.

Compound	Complex 1	Complex 2	1a
Formula	C_40_H_52_Bi_2_Cl_6_N_12_S_4_	C_40_H_52_Bi_2_Br_6_N_12_S_4_	C_40_H_52_Bi_2_Br_3.44_Cl_2.56_N_12_S_4_
Formula weight	1459.83	1726.59	1612.78
Crystal system	Triclinic		
Space group	P-1		
a (Å)	8.7586(4)	8.9058(2)	8.8574(4)
b (Å)	9.9283(4)	10.0482(2)	9.9894(4)
c (Å)	15.6437(6)	15.7157(4)	15.6899(7)
α (°)	102.728(3)	103.998(2)	103.389(3)
β (°)	91.557(3)	93.442(2)	92.605(3)
γ (°)	101.728(3)	100.0674(19)	100.859(3)
V (Å^3^)	1295.52(10)	1335.85(5)	1320.55(10)
Z	1	1	1
D_x_ (g cm^−3^)	1.871	2.146	2.028
F (000)	708	816	770
μ (mm^−1^)	7.296	11.263	9.586
Reflections:			
Collected	19,605	20,158	16,523
Unique (R_int_)	5717 (0.0361)	5938 (0.0335)	5237 (0.0258)
With I > 2σ (I)	5463	5581	5050
R (F) [I > 2σ (I)]	0.0260	0.0239	0.0168
wR (F^2^) [I > 2σ (I)]	0.0659	0.0264	0.0373
R (F) [all data]	0.0278	0.0576	0.0180
wR (F^2^) [all data]	0.0669	0.0592	0.0377
Goodness of fit	1.098	1.082	1.081
max/min Δρ (e·Å^−3^)	2.40/−1.68	1.06/−1.29	0.65/−1.00
CCDC deposition number	2312528	2312532	2312529
			
			
Compound	**1**b	**1**c	**2**a_n
Formula	C_40_H_52_Bi_2_Br_4.79_Cl_1.21_N_12_S_4_	C_40_H_52_Bi_2_Br_5.50_Cl_0.50_N_12_S_4_	C_40_H_52_Bi_2_Br_5_ClN_12_S_4_
Formula weight	1672.80	1704.36	1681.69
Crystal system	triclinic		
Space group	P-1		
a (Å)	8.8597(4)	8.8969(4)	8.8808(3)
b (Å)	10.0050(6)	10.0425(4)	10.0090(5)
c (Å)	15.7050(10)	15.7120(7)	15.6969(8)
α (°)	103.708(5)	103.879(4)	103.770(4)
β (°)	93.049(4)	93.256(3)	93.139(4)
γ (°)	100.409(4)	100.269(3)	100.395(4)
V (Å^3^)	1323.47(13)	1333.70(10)	1325.93(11)
Z	1	1	1
D_x_ (g cm^−3^)	2.099	2.122	2.106
F (000)	794	807	798
μ (mm^−1^)	10.516	10.932	10.637
Reflections:			
Collected	9857	16,523	12,260
Unique (R_int_)	4654 (0.0563)	4694 (0.0330)	12,260 (0.0566)
With I > 2σ (I)	4077	4459	9290
R(F) [I > 2σ (I)]	0.0435	0.0251	0.0351
wR (F^2^) [I > 2σ (I)]	0.0519	0.0614	0.0908
R (F) [all data]	0.1130	0.0261	0.0487
wR (F^2^) [all data]	0.1163	0.0624	0.0934
Goodness of fit	1.020	1.093	0.932
max/min Δρ (e·Å^−3^)	3.30/−2.20	1.94/−1.93	1.94/−1.16
CCDC deposition number	2312530	2312531	2312533
			
			
Compound	**2**a_p	**2**b	**2**c
Formula	C_40_H_52_Bi_2_Br_5.13_Cl_0.87_N_12_S_4_	C_40_H_52_Bi_2_Br_4.78_Cl_1.22_N_12_S_4_	C_40_H_52_Bi_2_Br_3.91_Cl_2.09_N_12_S_4_
Formula weight	1687.91	1672.35	1633.67
Crystal system	triclinic		
Space group	P-1		
a (Å)	8.8939(3)	8.8949(4)	8.8653(2)
b (Å)	10.0291(3)	10.0308(3)	9.9956(2)
c (Å)	15.7071(5)	15.7134(6)	15.6911(5)
α (°)	103.775(3)	103.726(3)	103.457(2)
β (°)	93.106(3)	93.010(3)	92.805(2)
γ (°)	100.389(3)	100.505(3)	100.7071(19)
V (Å^3^)	1331.50(8)	1332.38(9)	1322.54(6)
Z	1	1	1
D_x_ (g cm^−3^)	2.105	2.084	2.051
F (000)	800	794	778
μ (mm^−1^)	10.691	10.439	9.903
Reflections:			
Collected	9857	15,893	12,260
Unique (R_int_)	5435 (0.0348)	5262 (0.0404)	5869 (0.0221)
With I > 2σ (I)	5168	5001	5647
R (F) [I > 2σ (I)]	0.0265	0.0268	0.0195
wR (F^2^) [I > 2σ (I)]	0.0702	0.0676	0.0489
R (F) [all data]	0.0283	0.0288	0.0209
wR (F^2^) [all data]	0.0722	0.0695	0.0496
Goodness of fit	1.093	1.126	1.054
max/min Δρ (e·Å^−3^)	1.52/−1.98	1.28/−1.59	1.17/−1.05
CCDC deposition number	2312534	2312535	2312536

## Data Availability

Crystallographic data for the structural analysis has been deposited with the Cambridge Crystallographic Data Centre, Nos. CCDC—2312528—2312536. Copies of this information may be obtained free of charge from The Director, CCDC, 12 Union Road, Cambridge, CB2 1EZ, UK. Fax: +44(1223)336-033, e-mail: deposit@ccdc.cam.ac.uk, or www: www.ccdc.cam.ac.uk.

## Supplementary Materials

The following supporting information can be downloaded at: https://www.mdpi.com/article/10.3390/ijms251910814/s1.
