# Supplementary material for: Establishing Composition of Solid Solution Based on Single Crystal and Powder X-ray Measurement: The Case of Halogenated Bismuth(III) Complexes with Acetophenone-4-methyl-3-thiosemicarbazone
Source: Int J Mol Sci. 2024 Oct 8;25(19):10814. doi: 10.3390/ijms251910814 (PMC11477303; doi:10.3390/ijms251910814)
Supplement: Supplementary file 1 [file ijms-25-10814-s001.zip › ijms-3230398-supplementary.pdf]

Supplementary material for the paper:

Establishing composition of solid solution based on single crystal and powder X-ray measurement: The case of halogenated bismuth(III) complexes with acetophenone-4-methyl-3-thiosemicarbazone

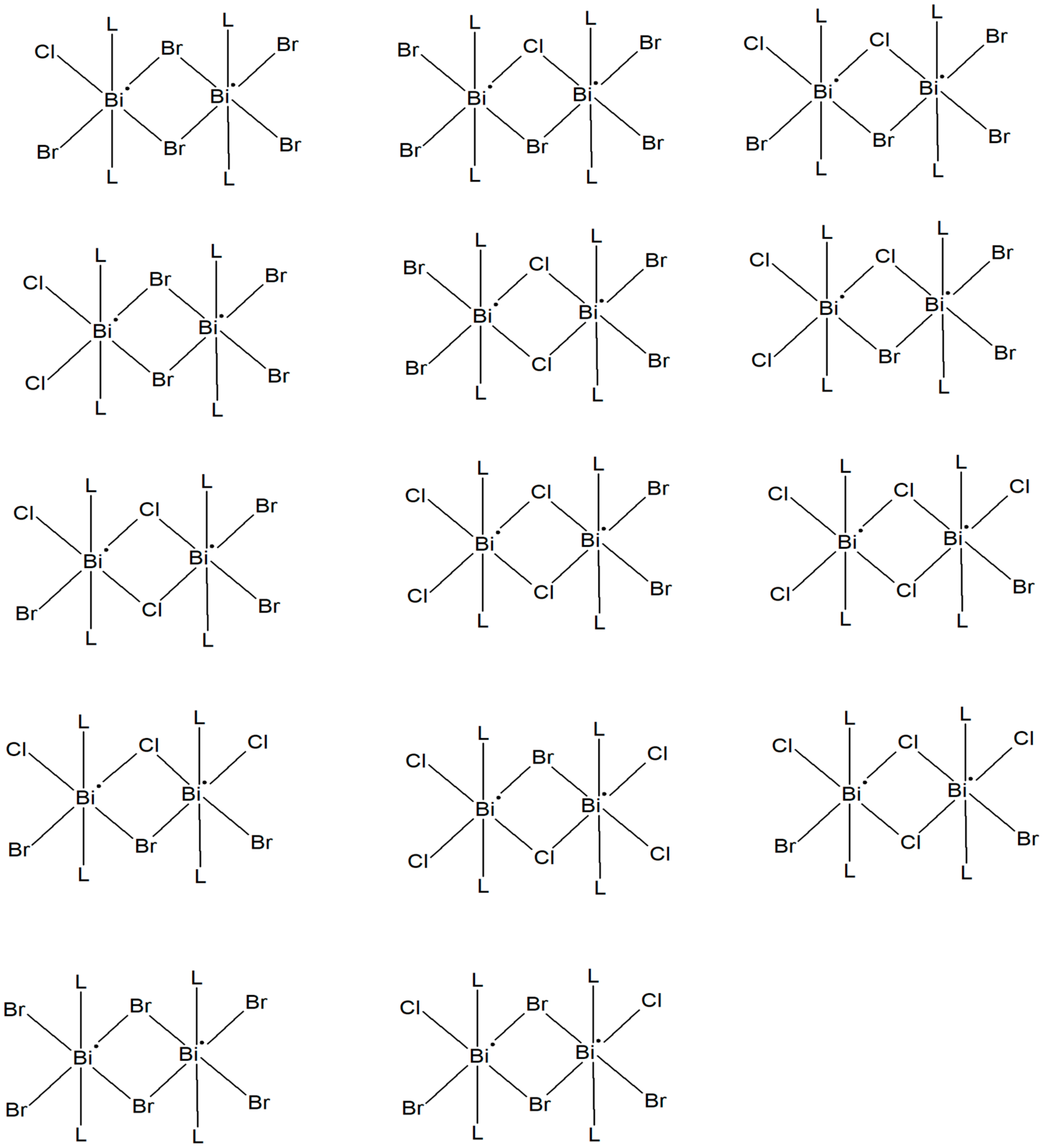

Fig. S1. Possible components of solid solution (limited to the same orientation of the ligand L)

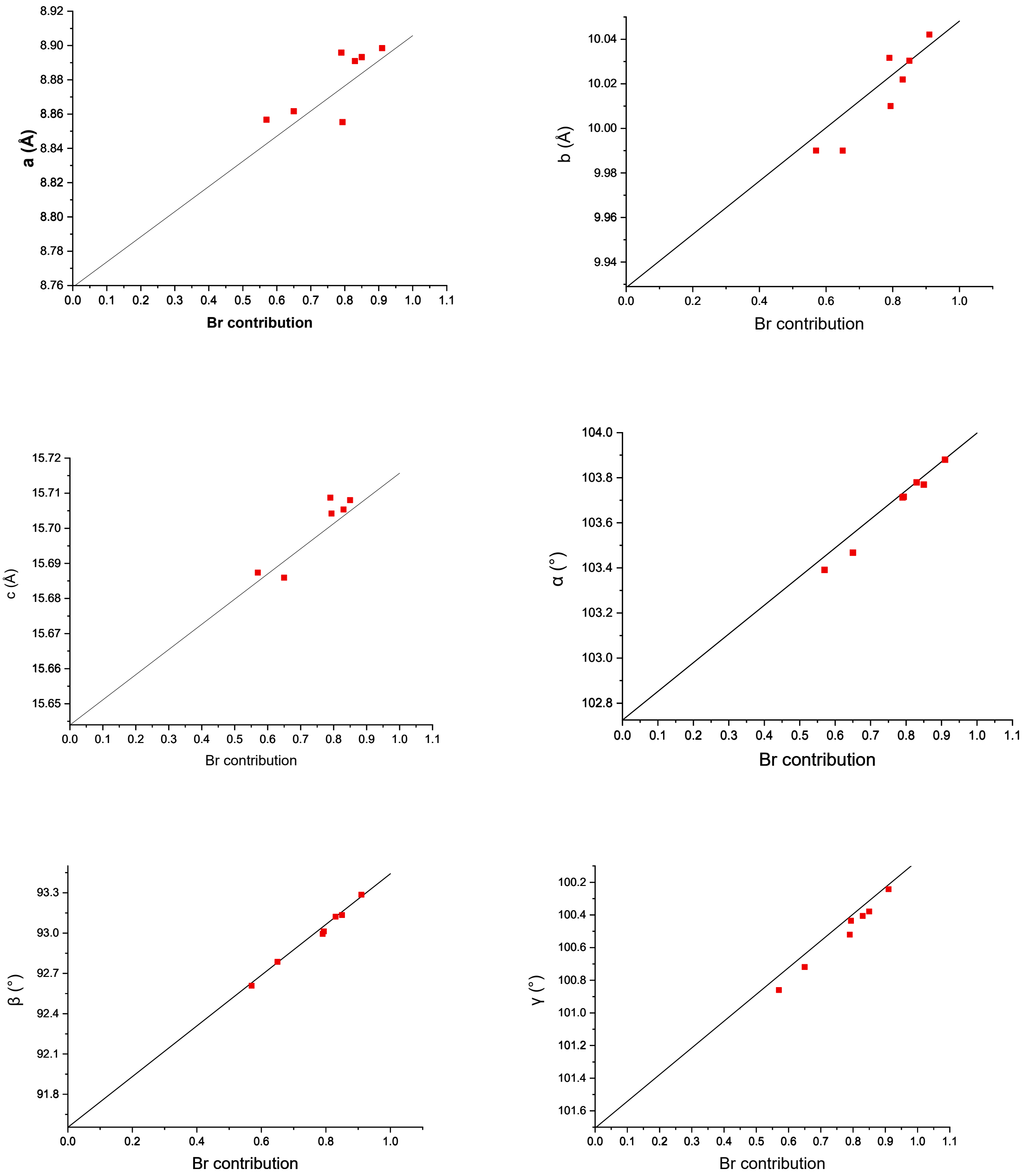

Fig.S2. Graphical representation of the deviation from the Vergard's law calculated for selected parameters

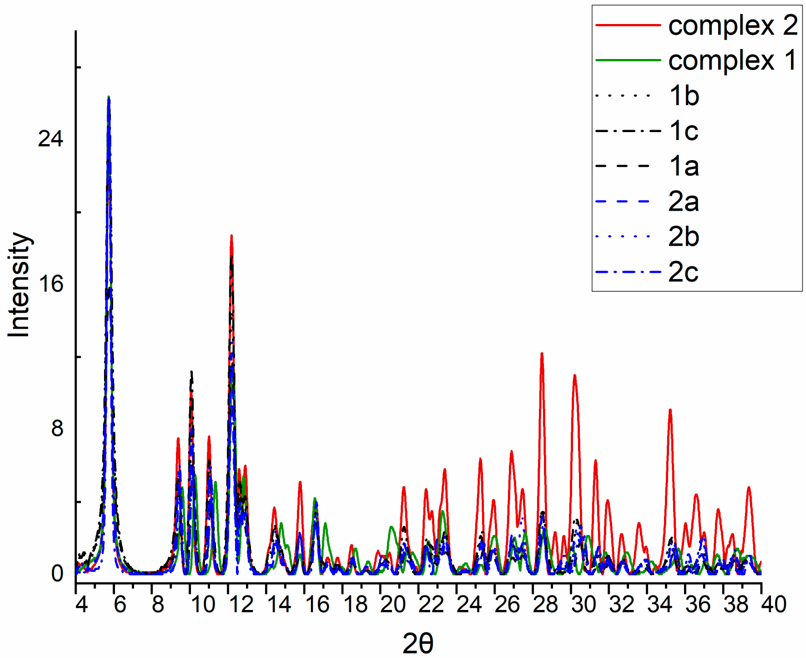

complex 2
complex 1
1b
1c
1a
2a
2b
2c
Intensity
0
8
16
24
4
6
8
10
12
14
16
18
20
22
24
26
28
30
32
34
36
38
40
2θ

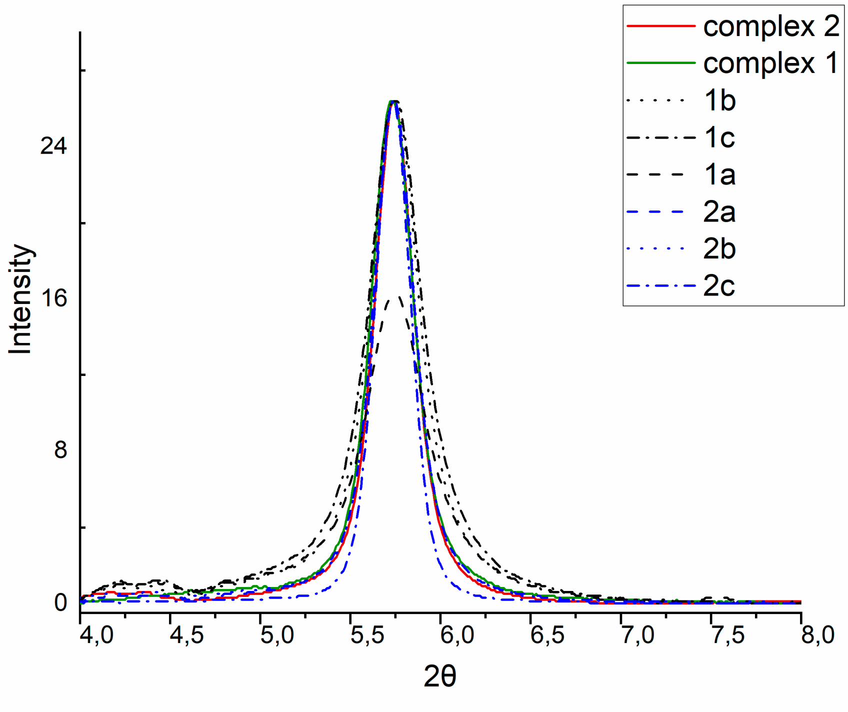

complex 2
complex 1
1b
1c
1a
2a
2b
2c
Intensity
0
8
16
24
4,0
4,5
5,0
5,5
6,0
6,5
7,0
7,5
8,0
2θ

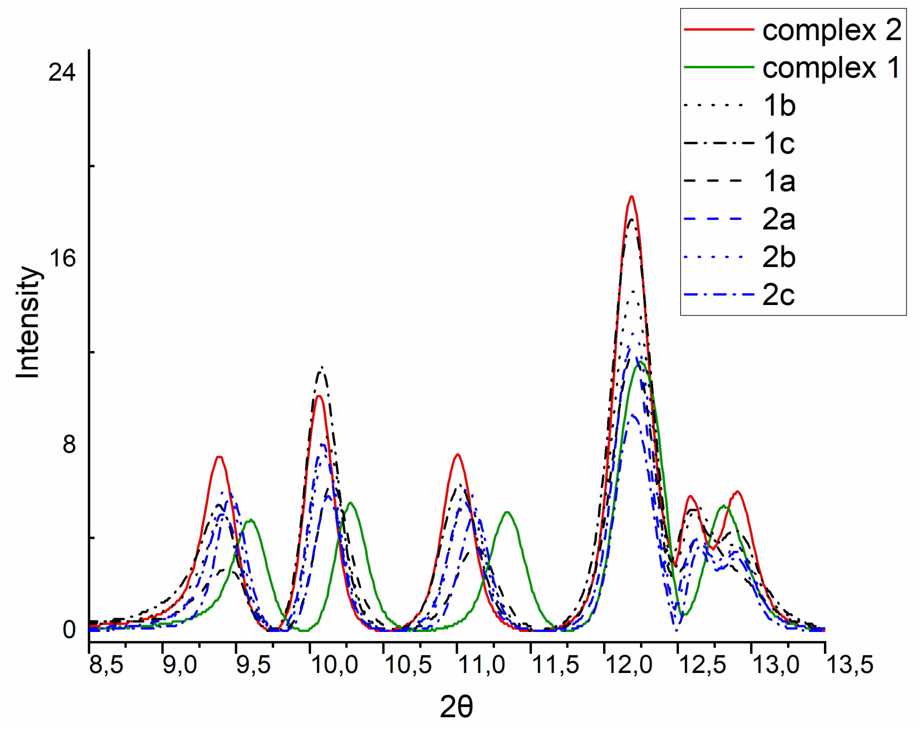
complex 2
complex 1
1b
1c
1a
2a
2b
2c
24
16
8
0
Intensity
8,5
9,0
9,5
10,0
10,5
11,0
11,5
12,0
12,5
13,0
13,5
2θ

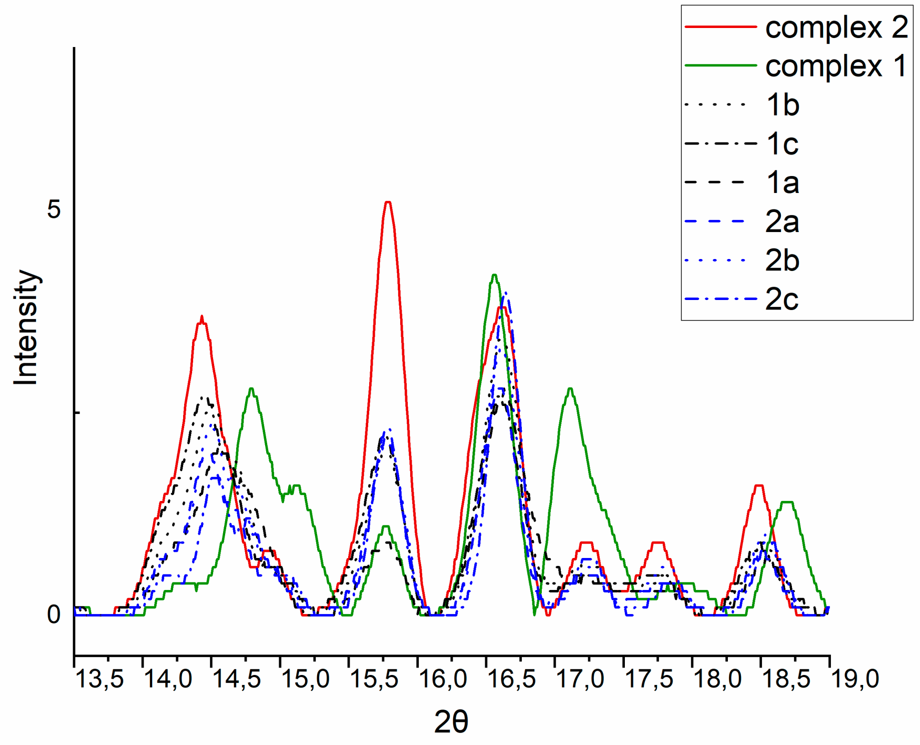
complex 2
complex 1
1b
1c
1a
2a
2b
2c
5
0
Intensity
13,5
14,0
14,5
15,0
15,5
16,0
16,5
17,0
17,5
18,0
18,5
19,0
2θ

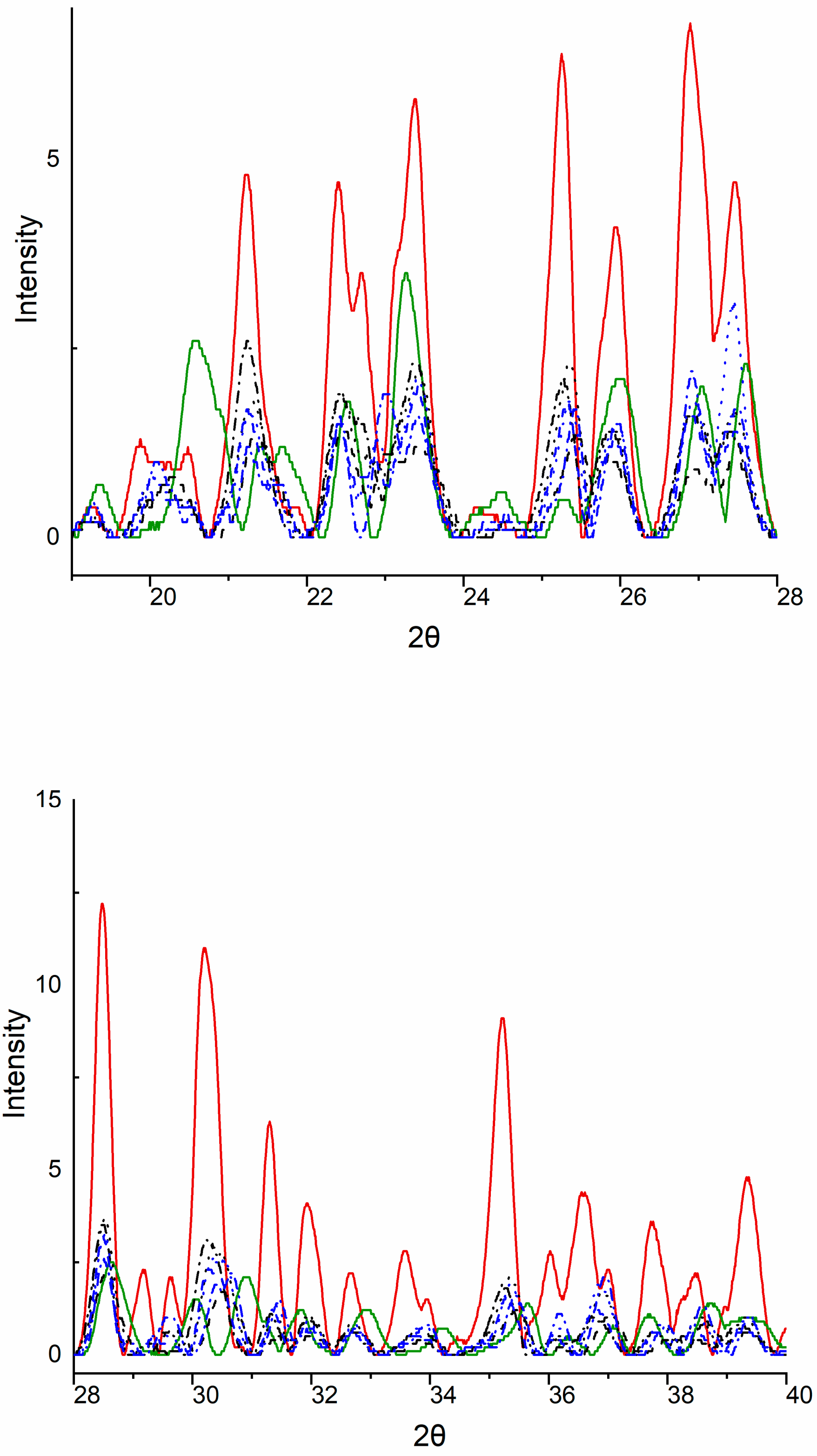

Fig. S3. Superposition of powder diffractograms for complex 1 (green) complex 2 (red) and solid solutions(blue and black)in selected 2θ angle ranges

Table S1. Relevant geometrical parameters (Å, °) with s.u.'s in parentheses. Symmetry codes: ^{i} 1-x, -y, 1-z; ^{ii} 1-x, 2-y, 1-z.

**1**		**2**	
Bi1-Cl1	2.8150(10)	Bi1-Br1	2.7712(4)
Bi1-Cl1^{i}	2.8874(9)	Bi1-Br2	2.7477(3)
Bi1-S10	2.8218(9)	Bi1-S10	2.8067(9)
Bi1-Cl2	2.5871(9)	Bi1-S30	2.8116(9)
Bi1-S30	2.7956(10)	Bi1-Br3	2.9483(4)
Bi1-Cl3	2.6277(10)	Bi1-Br3^{ii}	2.9868(3)
Cl2-Bi1-Cl1^{i}	165.73(3)	Br2-Bi1-Br3^{ii}	169.222(11)
S10-Bi1-S30	169.50(3)	S10-Bi1-S30	170.27(2)
Cl1-Bi1-Cl3	171.07(3)	Br1-Bi1-Br3	171.925(11)

Table S2. Occupancy in % calculated for bromine atom in three symmetry independent positions (data collected for two different crystals).

	Bridging	T1	T2	average	Bridging	T1	T2	average	Δ Av
**1**a	40.98	63.00	68.30	57.43	42.19	65.91	63.17	57.09	0.34
**1**b	69.92	83.26	85.81	79.66	70.63	83.32	83.17	79.04	0.62
**1**c	87.8	93.03	93.97	91.6	88.99	93.63	93.37	92.00	0.40
**2**a_p	80.00	88.15	88.53	85.53	78.04	87.52	89.72	85.09	0.47
**2**a_n	76.28	86.27	86.59	83.05	76.76	86.71	87.60	83.69	0.64
**2**b_n	73.9	82.44	84.37	80.24	71.50	81.54	82.79	71.50	1.63
**2**b_p	69.88	83.29	85.74	79.64	69.18	82.28	85.61	69.18	0.61
**2c**	51.38	70.0	74.23	65.93	53.65	70.14	74.02	65.94	0.73

Table S3. Occupancy in % calculated for bromine atom base on different parameters of the unit cell

	a	b	c	α	β	γ	V	**Avarage of occupancies[1]**	**Occupancies of average[2]**	OF
**1a**	66	51	69	53	55	52	65	63	58	57
**1b**	65	68	93	78	77	78	78	81	76	79
**1c**	98	99	112	90	90	87	103	100	96	91
**2a_n**	90	78	94	83	83	79	87	92	85	83
**2a_p**	91	85	98	82	83	81	93	91	87	85
**2a_av**	91	82	96	83	83	81	93	91	86	84
**2b**	93	86	99	78	76	72	96	88	84	79
**2c**	70	51	67	59	65	60	66	69	62	66

[1] The average of occupancies calculated for each parameter separately (a.b.c. α . β . γ)

[2] The occupancy calculated form average value of all unit cell parameter (a.b.c. α . β . γ)

Table S4. The 2θ angle position in theoretical and experimental powder diffractograms Please be attentive that theoretical and experimental data cannot be compered directly in terms of 2 θ angle values but only trends due to the different temperatures they have been measure in.

	**2 θ position**							
hkl	**(1)**	**(2)**	**1a**	**1b**	**1c**	**2a**	**2b**	**2c**
Theory 100K								
01-1	9.81	9.58	9.65	9.63	9.59	9.62	9.68	9.63
100	10.34	10.14	10.20	10.19	10.15	10.17	10.20	10.16
10-1	11.48	11.15	11.27	11.24	11.17	11.20	11.25	11.19
10-2	14.98	14.63	14.77	14.70	14.65	14.68	14.74	14.68
Experimental (room temperature)								
01-1	9.39	9.60	9.44	9.43	9.39	9.41	9.43	9.46
100	10.06	10.28	10.15	10.11	10.076	10.08	10.10	10.12
10-1	11.01	11.34	11.13	11.06	11.02	11.04	11.07	11.12
10-2	14.43	14.79	14.57	14.50	14.43	14.47	14.50	14.53

Table S5. Bromine concentration calculated based on each individual reflex shift

	Bromine concentration in %					
hkl	**1a**	**1b**	**1c**	**2a**	**2b**	**2c**
01-1	100.00	90.48	80.95	80.95	66.67	77.62
100	92.73	89.09	79.09	81.82	70.91	60.00
10-1	95.81	88.92	82.63	80.84	66.77	64.37
10-2	100.00	89.72	81.39	80.56	72.22	62.22
